# Unilateral pulmonary agenesis with ipsilateral facial congenital anomalies in an adult with obstructive airway disease

**DOI:** 10.1002/rcr2.1332

**Published:** 2024-03-31

**Authors:** Nilanthi Gunathilaka, Harshana Bandara, Tharindi Dissanayaka, Sathiaruban Sathiyamoorthy, Wathsala Gunasinghe, Saman Kularatne

**Affiliations:** ^1^ Pulmonology Department National Hospital for Respiratory Diseases Welisara Sri Lanka; ^2^ North West Lung Centre Wythenshawe Hospital Manchester United Kingdom

**Keywords:** facial anomalies, obstructive airway disease, pulmonary agenesis

## Abstract

Pulmonary agenesis is a rare congenital anomaly which can be isolated or co‐exist with other developmental defects. Boyden et al has described three degrees of mal development of lung which include agenesis, hypoplasia and aplasia. Almost all the reported cases are in paediatric age group patients while, adults with pulmonary developmental abnormalities are sparsely documented in the literature. Interestingly, adult with coexisting hemifacial anomaly and pulmonary agenesis has not been reported in the medical literature. Here, we describe a middle‐aged female who initially presented with bronchial asthma and her chest radiography showed absent left lung which was later confirmed with enhanced CT imaging. Furthermore, she had ipsilateral hemi facial microsomia, microtia, facial nerve palsy and mixed sensory loss, left side large café au lait patch, splenicule and hemangioma in segment V/VI of the liver.

## Graphical abstract

Lung agenesis is a rare entity to diagnose in adult pulmonology. Here we present an adult who presented with asthma with unilateral lung agenesis along with ipsilateral facial deformity.

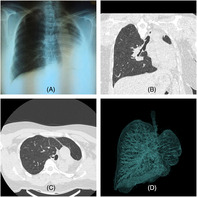

## INTRODUCTION

Pulmonary agenesis is a rare congenital anomaly which can be isolated or associated with other developmental defects.^1^, ^2^ Though the morphology of lung is preserved, there is an incomplete development of the lung tissue with regard to number and size of airways, alveoli and vessels.^3^ Here, we describe a middle–aged lady who presented to us with asthma exacerbation. Subsequently, she was found to have left pulmonary agenesis, ipsilateral hemi facial microsomia, microtia, facial nerve palsy and mixed sensory loss, left side large café au lait patch, splenicule and hemangioma in segment V/VI of the liver.

## CASE REPORT

A 48‐year‐old female was admitted to our care with exacerbation of underlying obstructive airway disease. She had been historically diagnosed as bronchial asthma and frequent chest infections since childhood. She had congenital hearing impairment in left ear and difficulty in closing her left eye. She is born to a non‐consanguineous parent and her family history is insignificant for syndromic births. Her birth was via uncomplicated vaginal delivery. However, according to the patient's knowledge, her intrauterine life has been complicated with exposure to attempts of termination of pregnancy with herbal remedies, using intra‐uterine Macrotyloma uniflorum (Horse gram). She was not aware of her early life development and health related problems. Despite her illness, she was occupied and has given birth to two babies without similar anomalies.

**FIGURE 1:**
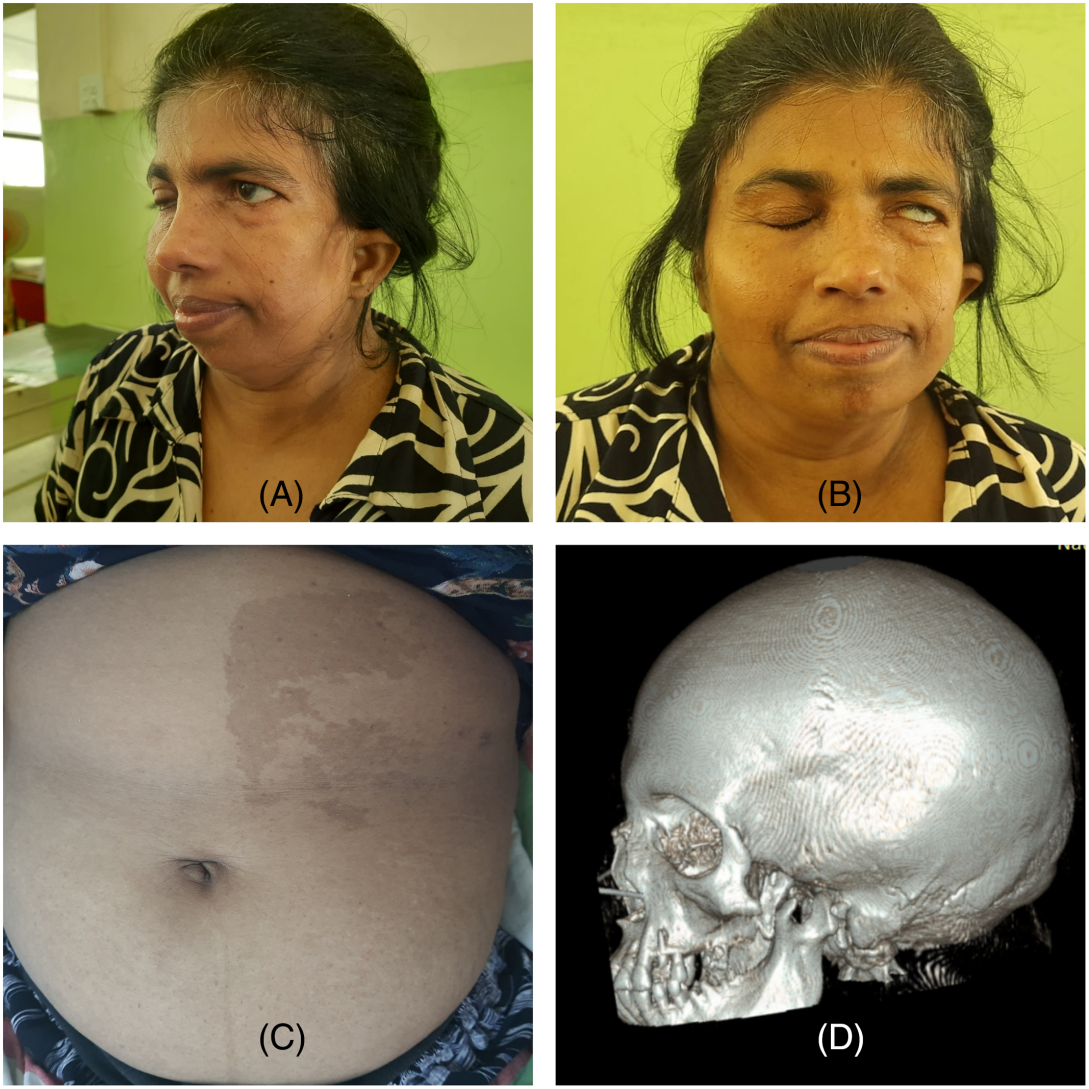
(A, B) Left hemi facial muscle atrophy, microtia (C) café au lait patch in anterior abdominal wall, (D) absent left zygomatic arch in lateral view of skull CT construction.

On examination, she had left side hemi facial microsomia with facial weakness over the distribution of upper branches of facial nerve and deformed left auricle. She had a flattened left hemithorax with mild degree scoliosis. A large café au lait patch was observed over the anterior abdominal wall without other neuro‐cutaneous or limb anomalies (Figure 1A–C). Her vital signs were stable while chest wall movements were reduced on left side with reduced breath sounds and expiratory wheezing on right chest.

**FIGURE 2:**
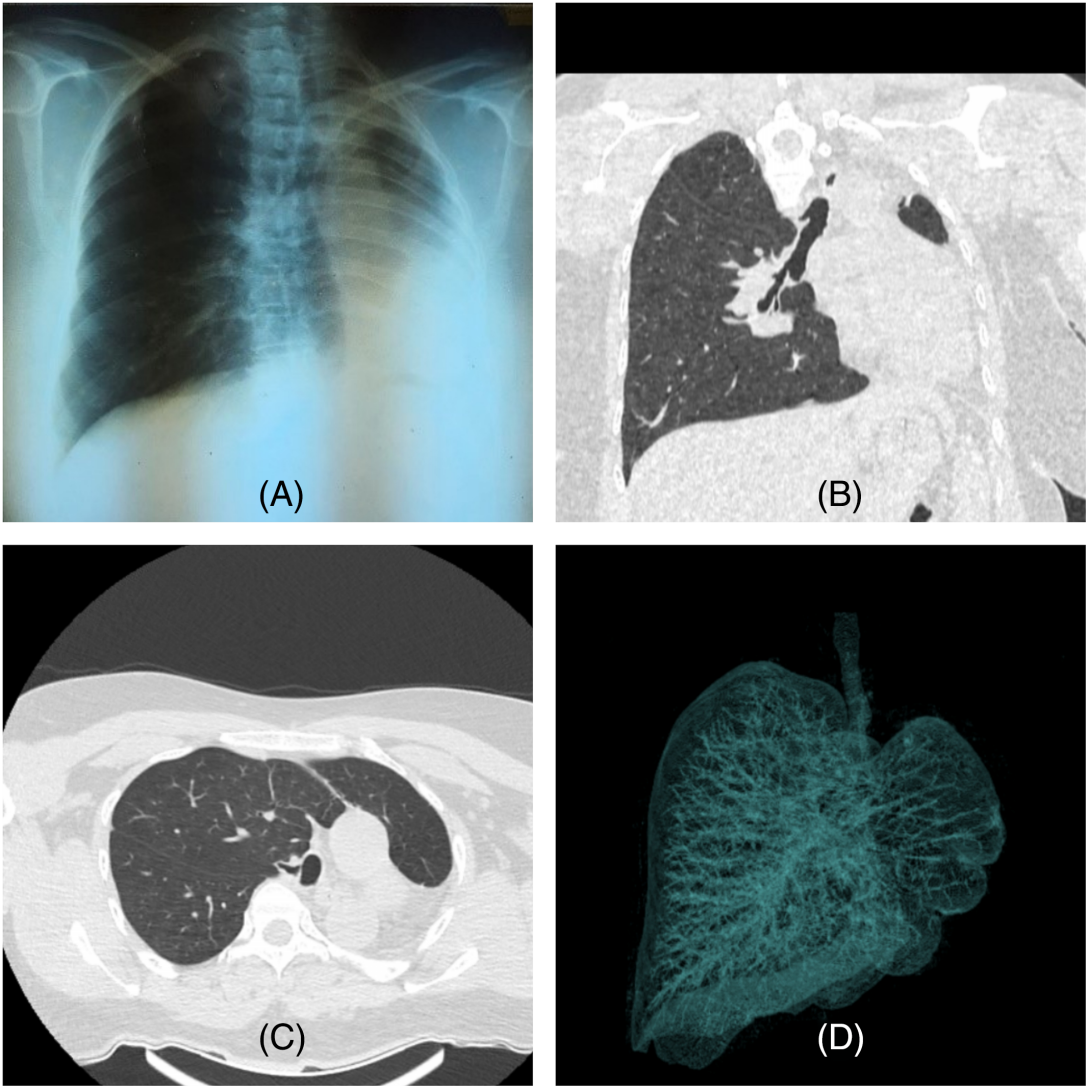
(A) Chest x‐ray showing left side homogenous opacity, hyperinflation of right lung, mediastinum shifting to left side and ipsilateral rib crowding. (B) Coronal imaging; absent left main bronchus and left lung agenesis. (C) Axial imaging; red arrow indicates the origin of right main bronchus from the trachea. (D) 3D imaging demonstrates absent left lung.

Patient's chest x‐ray showed left side homogenous opacity, hyperinflation of right lung, mediastinum shifting to left side and rib crowding of left hemi thorax. Computed tomography (CT) scan thorax revealed hypertrophied right lung with absent left pulmonary artery, bronchial tree and lung with mediastinal structures shifted to occupy the left hemi thorax (Figure 2). Her skull bones showed absent left zygomatic bone and atrophy of left side facial muscles (Figure 1D). CT abdomen showed splenicule and rounded lesion in segment V/VI of the liver. CT brain examination did not reveal internal capsular or facial nerve nuclear lesions.

Her pulmonary function test which was performed in a local hospital revealed obstructive airway disease with bronchodilator reversibility. She did not have airway eosinophilic inflammation as evidenced by normal serum IgE level, absence of sputum eosinophilia and low fractional exhaled nitric oxide (FeNO). Bronchial provocation test and lung volumes could not be performed due to the unavailability.

Audiogram revealed severe left side mixed hearing loss and right side mild to severe sensory neural hearing loss. Her cardiac, neurology and genitourinary screening was negative clinically and sonographically for anomalies.

Patient was acutely managed as bronchial asthma exacerbation and asthma control and reliever therapy with inhaled corticosteroids were prescribed. Otolaryngology opinion was sought for hearing impairment. Patient was reassured with regard to associated skeletal abnormalities, presence of splenicule and follow up was arranged in gastroenterology unit for hepatic hemangioma. Prevention of chest infections and regular vaccinations were encouraged.

## DISCUSSION

Structural alterations in respiratory system is a rare condition and typically can be detected in early life. Detection of pulmonary agenesis is extremely rare in adults.

Normal lung maturity occurs at the 4th and 24th week of gestation.^4^ Developmental abnormalities in broncho‐pulmonary foregut will lead into pulmonary hypoplasia or agenesis. Pulmonary arteries originate from the sixth aortic arch of the embryo. Involution of the proximal area of sixth aortic arch lead to pulmonary artery agenesis and hence the lung agenesis.^5^

Structural anomalies of lungs can vary from pulmonary hypoplasia to total lung agenesis. Pulmonary hypoplasia can be either primary or secondary. While primary hypoplasia is rare, secondary hypoplasia is well known to be associated with other congenital anomalies.^6^

In each one in 15,000 live birth, unilateral pulmonary hypoplasia has been estimated.^7^ The estimated incidence of total pulmonary agenesis is extremely rare (0.0034%–0.0097%).^8^ Though there is no specific predilection to either side,^1^ right pulmonary hypoplasia or agenesis is increasingly associated with other congenital abnormalities involving cardiovascular system (tetralogy of Fallot),^2^ central nervous system (anencephaly and hydrocephaly), gastrointestinal tract anomalies and other syndromes (Down syndrome, Klippel‐Feil syndrome).^9^

Pulmonary agenesis is rare in the literature and frequently associated with other congenital abnormalities. Most of the described cases are newborns and infants. Bilateral pulmonary agenesis is extremely rare and incompatible with life.

Boyden et al has described three degrees of mal development.^3^ Pulmonary agenesis is where there is complete absence of lung tissue. Aplasia describes the presence of rudimentary bronchus without lung tissue. Pulmonary hypoplasia is associated with all the normal pulmonary tissues, but they are underdeveloped. According to this classification, our patient belongs to the first degree of mal development.

Though pulmonary artery agenesis is commonly well tolerated these patients can manifest as recurrent chest infections, pleuritic chest pain, and hemoptysis or as other symptoms. Pulmonary agenesis association with bronchial asthma is also exceptionally rare.^10^

Hemi facial microsomia is a common facial anomaly which is encountered in 1 in 5000 births.^11^, ^12^ At the beginning of 4th week of embryogenesis facial structures are developed. Facial bones including maxilla, mandible, zygomatic bone, and temporal bone are developed from the first pharyngeal arch while external auditory meatus and the tympanic membrane are developed from the first pharyngeal cleft. Abnormal fusion of the first pharyngeal arch and the cleft will lead to hemi facial microsomia, microtia and external ear anomalies.

Diagnosis of pulmonary agenesis/hypoplasia relies on a high degree of clinical suspicion and requires a sound knowledge of chest x‐ray interpretation. Clinical presentation and the degree of respiratory compromise depends on the severity of anatomical abnormality. Even with advanced medical technologies, there are no effective treatment methods for pulmonary hypoplasia.

Fan et al. has described a child with hemifacial anomaly and lung hypoplasia.^13^ However there are no known hypothesis or relationship between congenital facial anomalies and pulmonary agenesis. However, in our patient, she had a potential teratogenicity following the attempted termination of the pregnancy using intra‐uterine Macrotyloma uniflorum (Horse gram). Wijenayake et al has described using animal studies that horse gram toxin exposure during early pregnancy lead to fetus implantation loss and mid‐pregnancy exposure leading to retarded organogenesis in the fetus.^14^ Therefore, we can assume that this exposure may have led to above mentioned lung agenesis and facial deformity.

Once a patient is detected with pulmonary hypoplasia or agenesis, other congenital anomalies should be excluded. Now a days during neonatal screening, most of the anomalies were detected early and corrective surgeries were taking place. In the past, due to lack of knowledge and socio‐economic barriers, it had led to uncorrected anomalies to persist and hence related sequale. Some of the congenital anomalies may not be lethal but uncorrected pulmonary anomalies can lead into chronic respiratory difficulties such as bronchial asthma, bronchiectasis, and recurrent respiratory infections.^15^

## AUTHOR CONTRIBUTIONS

Nilanthi Gunathilaka drafted the manuscript and Harshana Bandara revised the manuscript. All the authors approved the final manuscript.

## CONFLICT OF INTEREST STATEMENT

None declared.

## ETHICS STATEMENT

The authors declare that appropriate written informed consent was obtained for the publication of this manuscript and accompanying images.

## Data Availability

Data sharing not applicable to this article as no datasets were generated or analyzed during the current study.
